# Protein detection through different platforms of immuno-loop-mediated isothermal amplification

**DOI:** 10.1186/1556-276X-8-485

**Published:** 2013-11-18

**Authors:** Mohammad Pourhassan-Moghaddam, Mohammad Rahmati-Yamchi, Abolfazl Akbarzadeh, Hadis Daraee, Kazem Nejati-Koshki, Younes Hanifehpour, Sang Woo Joo

**Affiliations:** 1 Department of Clinical Biochemistry, Faculty of Medicine, Tabriz University of Medical Sciences, Tabriz 51656, Iran; 2 Department of Medical Biotechnology, Faculty of Advanced Medical Sciences, Tabriz University of Medical Sciences, Tabriz 51656, Iran; 3 Ian Wark Research Institute, University of South Australia, Mawson Lakes, South Australia 5095, Australia; 4 Department of Medical Nanotechnology, Faculty of Advanced Medical Sciences, Tabriz University of Medical Sciences, Tabriz 51656, Iran; 5 School of Mechanical Engineering, WCU Nanoresearch Center, Yeungnam University, Gyeongsan 712-749, South Korea

**Keywords:** iPCR, iRCA, iNASBA, ELISA, iLAMP, Protein detection

## Abstract

Different immunoassay-based methods have been devised to detect protein targets. These methods have some challenges that make them inefficient for assaying ultra-low-amounted proteins. ELISA, iPCR, iRCA, and iNASBA are the common immunoassay-based methods of protein detection, each of which has specific and common technical challenges making it necessary to introduce a novel method in order to avoid their problems for detection of target proteins. Here we propose a new method nominated as ‘immuno-loop-mediated isothermal amplification’ or ‘iLAMP’. This new method is free from the problems of the previous methods and has significant advantages over them. In this paper we also offer various configurations in order to improve the applicability of this method in real-world sample analyses. Important potential applications of this method are stated as well.

## Background

Proteins are biomolecules that have critical roles in living organisms. There are various types of proteins. Each type has a specific role in their corresponding organisms. The physiological status of organism determines the type and the concentration of a specific protein [1]. Also in the pathologic state, some variations occur in the type as well as concentration of proteins, depending on the type of pathological condition. For example, infectious diseases produce proteins that do not exist in healthy organisms [2]. Furthermore, in non-infectious diseases some proteins are produced not found in the healthy organism. These proteins are called biomarkers, and due to their specificity they can be applied in monitoring related disease. In addition to the disease-related proteins, other biologically important proteins need to be assayed [3].

Various methods are used for detection and quantification of biologically important proteins. Most of these methods are immunoassay-based techniques, which are divided into enzyme-substrate reactions, such as enzyme-linked immunosorbent assay (ELISA), and signal DNA amplification-based methods of protein detection, such as immuno-polymerase chain reaction (iPCR) [4,5]. ELISA is routinely used for assaying various proteins. The technique has some limitations. The most important limitation is its low sensitivity in detecting ultra-low-concentrated proteins [6,7]. On the other hand, signal DNA amplification-based methods have several advantages, including easy preparation of nucleic acids and specificity of sequence of signal DNA and its easy amplification [8]. For this reason, it has emerged as a powerful technique, known as ‘immuno-PCR’ or ‘iPCR’ through introduction of 100 to 10,000 times more sensitivity for detection of target proteins compared with routine ELISA [9]. Although iPCR have been designed to detect many proteins [10-19], it may suffer from important limitations including complicated protocol as well as requirement of special instruments and well-trained laboratory personnel. Therefore, it became necessary to design novel techniques to overcome the problems of iPCR [20]. Beyond iPCR other similar techniques have been proposed for detection of protein molecules with DNA as signal molecules. iReal-time PCR, immuno-rolling circle amplification (iRCA), and immuno-nucleic acid sequence-based amplification (iNASBA) are common examples of such methods. These methods have their own limitations as well, as discussed below.

In this study, we propose a new method for protein detection. The proposed method comprises of two main steps, including signal amplification step, called immuno-loop-mediated isothermal amplification (immuno-LAMP or iLAMP), followed by ultra-sensitive detection of amplified signal. Here we discuss the main aspects of this new technique while comparing it with current nucleic acid-based detection methods for proteins.

## The hypothesis and its evaluation

### Immuno-LAMP

Loop-mediated isothermal amplification (LAMP) is a new method developed in year 2000 by Notomi et al. Basically, this method of DNA amplification uses a specific DNA polymerase enzyme and a set of four specific primers that distinguish six different regions on the sequence of the target DNA. The primers consist of inner primer pair [FIP (forward inner primer) and BIP (backward inner primer)] and outer primer pair [F3 (forward outer primer) B3 (backward outer primer)]. Inner primers contain sequences of the sense and antisense strands of the target, while outer primers contain only the antisense sequence of the target strands. In the first step of LAMP, an inner primer starts the reaction and the newly produced strand is displaced by annealing of an outer primer on the same target strand and subsequent synthesis of complementary product strand. The displaced product strand (primed by inner primer) itself serves as template for synthesis of new strand primed by the second inner and outer primers, which hybridize to the other end of the target DNA; the strand adopts stem-loop structure. The next round of LAMP reaction produces a new stem-loop DNA structure, which has a stem twice as long. The reaction continues until accumulation of stem-loop DNA structures with several inverted repeats of the target and cauliflower-like structures with multiple loops formed by annealing between alternately inverted repeats of the target in the same strand. The reaction takes less than an hour, and produces 10^9^ copies of the target DNA [21].

Due to its significant advantages over PCR, since its development, LAMP has been found in many applications in the molecular diagnosis including detection of pathogens, genetically modified organisms, embryo sex detection, and tumor detection [22]. One advantage is that in LAMP method, a particular bacterial DNA polymerase, named Bst DNA polymerase, is used for amplification of the target in isothermal condition without application of any specific thermal cycler. Because this enzyme denatures simultaneously the substrate double-strand DNA and subsequently synthesizes products, there is no need for pre-denaturation of target dsDNA, especially by heat, which is used commonly in PCR technique. The amplification thus can be performed in a simple water bath or other heating tool in isothermal condition.

Secondly, in spite of PCR, products of LAMP can be detected visually by the naked eye through turbidity or adding DNA intercalating dyes (e.g., SYBR green) without any need for specified equipment (gel electrophoresis and UV gel documentation systems which are necessary in PCR). Also, since the amplification and detection are performed in the same tube, unlike PCR, LAMP can be performed in closed reaction tubes and it minimizes any cross-contamination risk while using multiple samples [21,23-25]. Besides this advantage, LAMP is not as sensitive as PCR toward inhibitors [26]. It thus contributes to lowering of LAMP costs compared with PCR.

The third advantage is the higher specificity and sensitivity of LAMP over PCR. Unlike common PCR, performed using one pair of primer, LAMP requires at least two pairs of primer and, thus, increases the chance of specific recognition and amplification of target DNA compared to PCR. Higher sensitivity of LAMP originates from the unique mechanism of amplification, known as loop-mediated amplification, which produces huge amplicons consisting of repeated loop-shaped and dumbbell-like DNA structures. This type of amplicons allows easier detection of positive results and lowers the limit for detection. Therefore, it increases sensitivity of LAMP in comparison with PCR. The fourth advantage of LAMP is its speed over PCR. The overall time for carrying out LAMP is about 1 h compared with 2 to 4 h in PCR [21,23-25] Moreover, the rate of LAMP reaction can be increased by using two additional primers, called loop primers [27]. Therefore, LAMP is considered a more rapid molecular technique compared with PCR.

LAMP can be performed in a high-throughput format for simultaneous analysis of large-number samples in 96-well microplate [28]. Lower cost, quickness, simplicity, specificity, and sensitivity make LAMP a powerful alternative for PCR in the field of molecular diagnosis, especially in developing countries. Therefore, considering the advantages of LAMP over PCR, it can be used in most of molecular methods that utilize PCR. One of the molecular methods, which can use LAMP instead of PCR, is ‘immuno-PCR’ or ‘iPCR’. iPCR is usually used for detection as well as quantification of antigens (Ags), which are mostly protein, using PCR. In this method target Ag is captured in a sandwich form between two antibodies (Abs), the capture antibody and the detection antibody, which are specifically bound to the target antigen. The capture Ab, which is pre-immobilized on a solid support surface, captures the target Ag, and the detection Ab, which is pre-conjugated with a double-strand DNA called signal DNA, attaches to the captured Ag. After wash, the signal DNA is amplified by PCR, and hence the presence of PCR products indicates indirectly the presence of target Ag in the sample. In fact, in iPCR, PCR is used for signal amplification. Since PCR method produces millions of copies of target DNA, iPCR converts the presence of a few Ag molecules into a signal, which is easily detectable. Thus, iPCR can detect Ag in very low quantities and is more sensitive than common Ag detecting methods like ELISA [9]. However, iPCR itself may have some technical limitations. Some practical drawbacks make this method difficult to be easily utilized in low-resource laboratories. These limitations include complicated and time-consuming protocol, requirement for specific tools and expert personnel for performing of the method, low signal-to-noise ratio, the risk of cross-contamination among different samples when assaying multiple samples, and technical hurdles in the preparation of detection of antibody-signal DNA conjugates. The real-time iPCR also requires advanced thermal cyclers and more specified reagents compared with iPCR [20].

iRCA is another version of nucleic acid-based method for protein detection. In this technique, a specific DNA polymerase enzyme is used to elongate the primer DNA, which hybridizes to a circular DNA as the template [8]. This technique has been used for detecting prostate-specific antigen [29], as well as simultaneous detection of cytokines' and allergens' specific antibodies in a microarray format [30-32], and introduced commercially for chip-based amplification [20]. Some disadvantages of iRCA are common with iPCR. These limitations include cumbersome preparation of antibody-signal DNA conjugates, complicated and time-consuming protocol, risk of cross-contamination among different samples, no quantification capacity of rolling circle amplification (RCA) reaction, complex primer design, and no tolerance to complex biological environment [33].

Another form of nucleic acid-based protein detection is known as iNASBA, which has been used for high-throughput detection of adrenocorticotropic hormone and two common waterborne pathogens, *Escherichia coli* and *Rotavirus*, in 96-well microplate format [34]. Nucleic acid sequence-based amplification (NASBA) technique has some limitations over LAMP method. Firstly, a suitable substrate for NASBA reaction is RNA, while that for LAMP is DNA. Instability of RNA makes LAMP more appropriate for application in protein targets. The second limitation of NASBA originates from the requirement of reverse transcriptase, RNase H, and T7 DNA-dependent RNA polymerase during reaction, but in LAMP only DNA polymerase is needed. The third limitation is that products of NASBA are RNA molecules and gel electrophoresis is necessary to detect them. This makes NASBA more liable and dependent on special equipments. There is no need for gel electrophoresis for detection of LAMP products, which are DNA molecules. Moreover, due to the instability of NASBA products, quantification of iNASBA is not easy in comparison with iLAMP products. Although the amplification reaction of NASBA is isothermal, a single melting step prior to the amplification reaction is required to allow annealing of the primers to the target, which is not required in LAMP [35,36].

**Figure 1:**
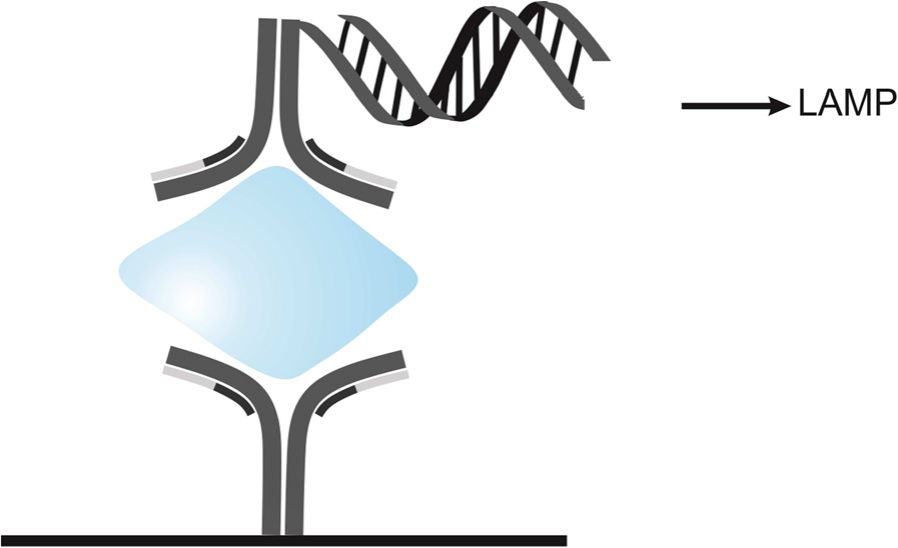
The principle of iLAMP reaction.

Considering the advantages of LAMP over PCR, RCA, and NASBA methods, one can believe that LAMP can be used instead for detection of Ags. We name this method ‘immuno-loop-mediated isothermal amplification’ or ‘iLAMP.’ This novel method can detect Ags, for instance proteins, with ultra-specificity and sensitivity as well as rapidness, low cost without the need for expert personnel, and advanced instruments in comparison with ELISA, iPCR, iRCA, and iNASBA [23]. In this novel technique, the target protein is first captured by specific antibody or aptamer, and the second specific antibody or aptamer recognizes the captured protein. This secondary antibody is pre-conjugated with a known DNA sequence that is amplified in the subsequent LAMP reaction after release from the secondary antibody. In the case of aptamer, it is possible to directly use it as the substrate for the LAMP due to the fact that it is nucleic acid and it can be easily amplified by LAMP reaction (Figure 1).

Table 1 summarizes the main features of the mentioned techniques and iLAMP.

**Table 1:** Comparison of different immunoassay methods used for protein detection

**Method**	**Required time**	**Complicacy**	**Requirement of high expertise and instrumentation**	**Cost**	**Specificity**	**Sensitivity**	**Quantification capacity**	**Potential of being as high-throughput test**	**Potential for point-of-care application**	**Risk of cross-contamination**
iLAMP	Low	Low	No	Low	High	High	Yes	Yes	Yes	Low
iPCR	High	High	Yes	High	Intermediate-high	Intermediate-high	No	No	No	High
iReal-time PCR	High	High	Yes	High	Intermediate-high	Intermediate-high	Yes	No	No	High
iRCA	High	High	Yes	High	High	Intermediate-high	Yes	Yes	No	High
iNASBA	High	High	Yes	High	High	Intermediate-high	Yes	Yes	No	High
ELISA	High	High	Yes	High	Low	Low	Yes	Yes	No	High

### Possible configurations of iLAMP

iLAMP can be modified as different platforms in order to enhance its capabilities. Amplification of signal DNA by LAMP is considered as the first step of signal amplification, which is achieved through performing LAMP followed by detection of LAMP products by common methods, such as turbidimetry, inspection by naked eye, and application of DNA intercalating dyes [24]. These methods can also be applied to the detection of iLAMP amplification product.

Sometimes further amplification of the signal may be necessary, particularly in the case of detecting trace proteins. In these cases, it can be achieved by enhancing the detection of LAMP products through more sensitive methods. Application of nanoprobes, integration with signal DNA-containing liposome, and microfluidic technology can increase the sensitivity and selectivity of iLAMP.

Also, some modifications can be implemented into iLAMP to improve its performance, such as integration with microfluidic technology and application of aptamers instead of antibodies for capturing as well as detection of target proteins. A number of potentially important modifications are discussed below.

#### Integration with nanoprobes

Nanoprobes are nanoscale tools, which are used for detecting and monitoring various molecular targets. In biological purposes, they can be designed to detect biomacromolecules, such as DNA, RNA and proteins. They are composed of sensor and detector part. Sensor part is used to signal the presence of target molecule, while the detector part recognizes the target molecule. This recognition is based on the specific interaction of target molecule with the detection part of the nanoprobe. For detection of DNA and RNA, the detector part is a strand of nucleic acid, which specifically hybridizes with target DNA or RNA molecule. Nanoparticle-based nanoprobes are excellent tools for detection of nucleic acids. They have a nanoparticle (as sensory part) and probe part (as detection part).

In regards to the fact that the product of iLAMP is DNA, molecular nanoprobes can be utilized to detect it. The application of nanoprobes adds further sensitivity and specificity to iLAMP. Considering the fact that the sequence of iLAMP products can be inferred from the sequence of signal DNA, nanoprobes can be easily designed for specific detection of iLAMP products. Application of these nanoprobes can have potential advantages. Firstly, application of probes makes this method more specific than other current methods. Secondly, color change can be easily quantified by simple spectrophotometry or colorimetry based on color intensities, so that color intensities indirectly can be correlated with concentration of target protein [37]. This format is called ‘iLAMP-nanoprobe’ method and can be an appropriate alternative for real-time iPCR, which is used for quantification or determination of the primary concentration of target protein. Real-time iPCR has significant pitfalls which iLAMP-nanoprobe method does not have. These pitfalls include high cost, requirement of expert personnel, advanced instruments, and much time [20]. Thus, in addition to qualitative iPCR and other similar methods, iLAMP-Au-nanoprobe method can be used instead of real-time iPCR.

Different nanoparticle-based nanoprobes have been designed so far. Gold, silver, and quantum dot (fluorescent) nanoparticles are main nanoparticles that are used for detection of target nucleic acids.

##### Gold nanoprobes (Au nanoprobes)

Gold-nanoparticle probes take the optical advantages of gold nanoparticles at the time of specific hybridization between their nucleic acid parts with target nucleic acids. The hybridization brings the gold nanoparticle part of these probes near each other, leading their aggregation and subsequent color change from deep red to blue/purple [38].

Generally, two main formats are used to detect target DNA by Au nanoprobes called ‘homogenous’ or ‘solution-based’ format and ‘heterogenous’ or ‘solid-based’ format. In the homogenous format, the hybridization of Au nanoprobes with target occurs homogenously without any attachment to any solid supports. However, in the heterogenous format the recognition of target sequence occurs on a specific probe sequence linked to a solid support. The routine method of target detection using heterogenous format is a sandwich-type reaction, in which target sequence is hybridized with two specific probes, so that one probe is attached to a solid base; after hybridization of target sequence, the secondary probe (Au nanoprobe) hybridizes with the other part of target sequence. The presence of specific target is detected then by the use of silver enhancement. This format of detection shows more sensitivity and specificity for recognition of target nucleic acid compared with homogenous format [38]. Although silver enhancement has some drawbacks, the results can be quantified based on the intensities of reduced silver. The drawbacks are high background of silver enhancement and weak signal-to-noise ratio [39]. However, these can be avoided by using gold enhancement instead of silver enhancement. Gold enhancement has been used in two studies for detecting target nucleic acid in homogenous format [40,41]; and due to the high false-positive results associated with silver enhancement [39], gold enhancement can be used for the detection of target nucleic acids in heterogenous format and can be applied for quantitative detection of iLAMP products by Au nanoprobes in iLAMP-Au-nanoprobe method. Another advantage of heterogenous format is its applicability for simultaneous, high-throughput assay of several samples. This can be achieved using 96-well or 384-well microplates so that each single well can be a site for one reaction.

Another type of solid-base format is the application of paper strips for detection of targets by Au nanoprobes [34]. Although this type does not allow quantification, it can be used to read out results of iLAMP-Au-nanoprobe method beside previously described homogenous and heterogenous formats.

Recently, some studies have shown the advantages of Au nanoprobes to detect LAMP product. In these studies, LAMP products were specifically hybridized with Au nanoprobes, and upon hybridization the color of reaction was changed from red to blue [42-44].

Accordingly, regarding the advantages and application of Au nanoprobes for detection of LAMP products, the aforementioned formats can be used for detecting iLAMP products in iLAMP-Au-nanoprobe method.

##### Silver nanoprobes (Ag nanoprobe)

Silver nanoparticles (Ag) have analogous properties to gold nanoparticles. Thus, they have been used in two recent studies to prepare Ag nanoprobes for the detection of target DNA molecules [45,46]. In these studies, the presence of target DNA was detected by spectral changes in surface plasmon resonance of gold nanoparticles and visual inspection. The advantage of Ag nanoprobes over Au nanoprobes is that due to the greater extinction coefficient of silver nanoparticles in comparison with gold nanoparticles, much lower concentrations of Ag nanoprobes are required to analyze spectral absorption, and thus the sensitivity of Ag nanoprobes are more than that of Au nanoparticles with the same concentration.

Like Au nanoparticles, silver/gold enhancement can also be applied at the time of target DNA detection with Ag nanoprobes in order to increase the sensitivity as well as to make the quantification because Ag and Au nanoparticles have similar optical properties [47].

##### Gold-silver alloy nanoprobes (Au-Ag nanoprobes)

Au-Ag nanoprobes have the optical properties of silver nanoparticles (high extinction coefficient) with ease of functionalization via a thiol bond provided by the gold at the same time. Preparation of the alloy nanoparticles solves the problems associated with the functionalization of Au and Ag composite nanoparticles while retaining the beneficial properties for DNA detection [48].

Moreover, it is possible to use Au-Ag nanoprobes and Au nanoprobes inside the same reaction to detect simultaneously two different targets. This capability is due to different optical properties of Ag-Au alloy nanoparticles with Au nanoparticles, which makes it possible to perform multiplex assays. Detection of more targets simultaneously can be possible through application of alloys with different Ag/Au ratios [48]. This property can be exploited in iLAMP method for designing multiplex assays that detect different protein targets simultaneously.

##### Quantum dot (fluorescent) nanoprobes

Quantum dots (QDs), the semiconductor nanocrystals with 100 to 100,000 numbers of atoms, have unique optical properties. They are relatively photostable in comparison with common fluorescent dyes. These properties make them attractive candidates for designing optical nanoprobes and, thus, are used in real-time and continuous detection of target molecules. The most common method of using QDs as nanoprobes is utilizing them in order to create an ‘on/off’ signal via Förster resonance energy transfer (FRET) phenomena whereby non-radioactive energy transfer occurs between the QD (donor) and an acceptor molecule (quencher), while monitoring molecular distances and interactions at the nanoscale level [49-51].

Different methods of target DNA detection has been used using FRET phenomena. Most of these methods are based on the hybridization between target DNA and QD-tagged probes (QD nanoprobes). These probes are usually double-tagged with QD (in one end) as well as a quencher molecule (in the other end). As mentioned previously, these nanoprobes can be designed to produce signal (signal on) or disappear the signal of tagged QD molecule (signal off) when they recognize target DNA. In the signal-on method the probe DNA is designed as stem-loop structure. In this state QD is located in the vicinity of quencher molecule, absorbing the fluorescent signal of QD and preventing it from detection. However, when the nanoprobe hybridizes with the target DNA, the stem-loop structure denatures and is converted to the linear conformation. The QD and quencher molecule are thus located away from each other, making it possible to detect the fluorescent signal of tagged QD.

In the signal-off method, the nanoprobe is tagged with QD and the target is tagged with quencher. In unhybridized state, the tagged QD emits signal and its signal is detectable. But when the nanoprobe recognizes the target DNA, it hybridizes the target DNA, leading to bringing QD to the vicinity of quencher and prevention of QD emission [52].

QDs can also be used as electroactive labels for detection of target DNA in the electrochemical biosensors. This property originates from inherent electrochemical properties with ease of miniaturization, low cost, low power requirements, and excellent biocompatibility. In electrochemical detection of target DNA molecules by QDs, they serve as electrochemical catalyst (electroactive molecules), which transports loads of electrons through the reduction of dissolved oxygen, resulting in a significant increase in the reduction peak current. In fact, they show sharp voltammetry signals proportional to the concentration of corresponding DNA targets and serve as signal-enhancing agents [53].

**Figure 2:**
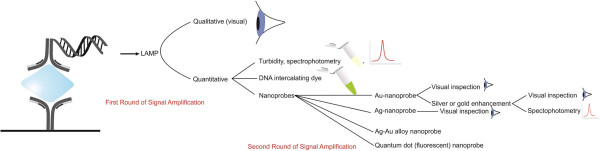
The principle and possible ways of iLAMP products analysis with different nanoprobes (nanoprobe-iLAMP platform).

In addition to detection of one target, several different-sized QD molecules can be excited by one excitation source simultaneously. This ability is advantageous in detecting more than one target in the same time [54]. It is thus possible to implement multiplex iLAMP assays for detecting multiple proteins in a sample. The application of nanoprobes for detecting iLAMP products is depicted in Figure 2.

#### Integration with liposome

Liposomes are spherical micro/nanostructures made of lipid bilayers and can be filled with various molecules. In other words, liposomes have hydrophilic cores, which can be loaded with hydrophilic agents [55]. They can be loaded with thousands of DNA molecules as signal molecules, and at the time of detection liposome membrane can be destructed in order to release the signal DNA molecules. Signal DNA molecules released then can be readily amplified with LAMP method. Application of DNA-loaded liposomes instead of single signal DNA increases the sensitivity of iLAMP drastically. Releasing several molecules of signal DNA from liposomes increases the possibility of recognition of target signal DNA by LAMP enzyme (Bst DNA polymerase). In fact, DNA-loaded liposomes serve as the first step of signal amplification, and LAMP serves as the second. Furthermore, application of nanoprobes for detection of LAMP products adds the third step of signal amplification to the iLAMP reaction. It can enhance the sensitivity of iLAMP several times. This significant increase of sensitivity can be useful for detection of very low concentration proteins and detection of target proteins in complex samples by overcoming the inhibition of Bst DNA polymerase by inhibitors existing in the sample.

The application of DNA-encapsulated liposome has been reported in a study, where a modified version of iPCR, called as immunoliposome-PCR, has been utilized to measure the concentration of carcinoembryonic antigen (CEA) in human serum. This study showed that this novel method is 1,500 times more sensitive than common methods of CEA detection [56]. Similarly, immunoliposome-LAMP method can be designed to considerably enhance the detection limit of iLAMP.

**Figure 3:**
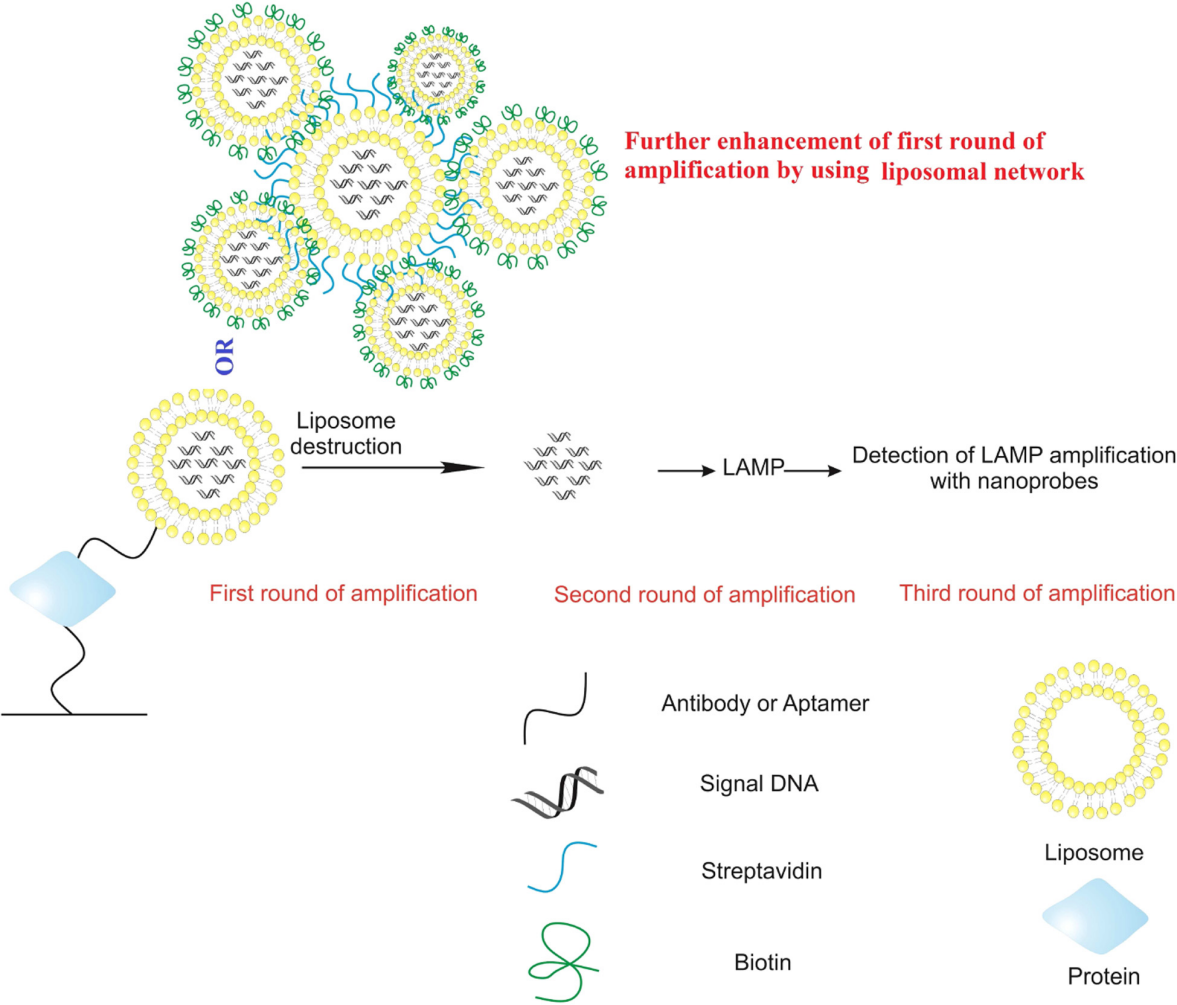
Integration of liposome with iLAMP (liposome-iLAMP platform).

More layer of signal enhancement in immunoliposome-LAMP can be reached through application of liposomal networks, instead of application of one liposome for detection of target protein. Practically, this network can be constructed through application of biotin-streptavidin interactions. For construction of liposomal network, biotin-embedded liposomes, pre-loaded with signal DNA molecules, can be linked to each other through streptavidin or avidin bridges. This improvement increases the sensitivity of iLAMP significantly in comparison with single-immunoliposome-LAMP (Figure 3).

#### Integration with microfluidic devices

**Figure 4:**
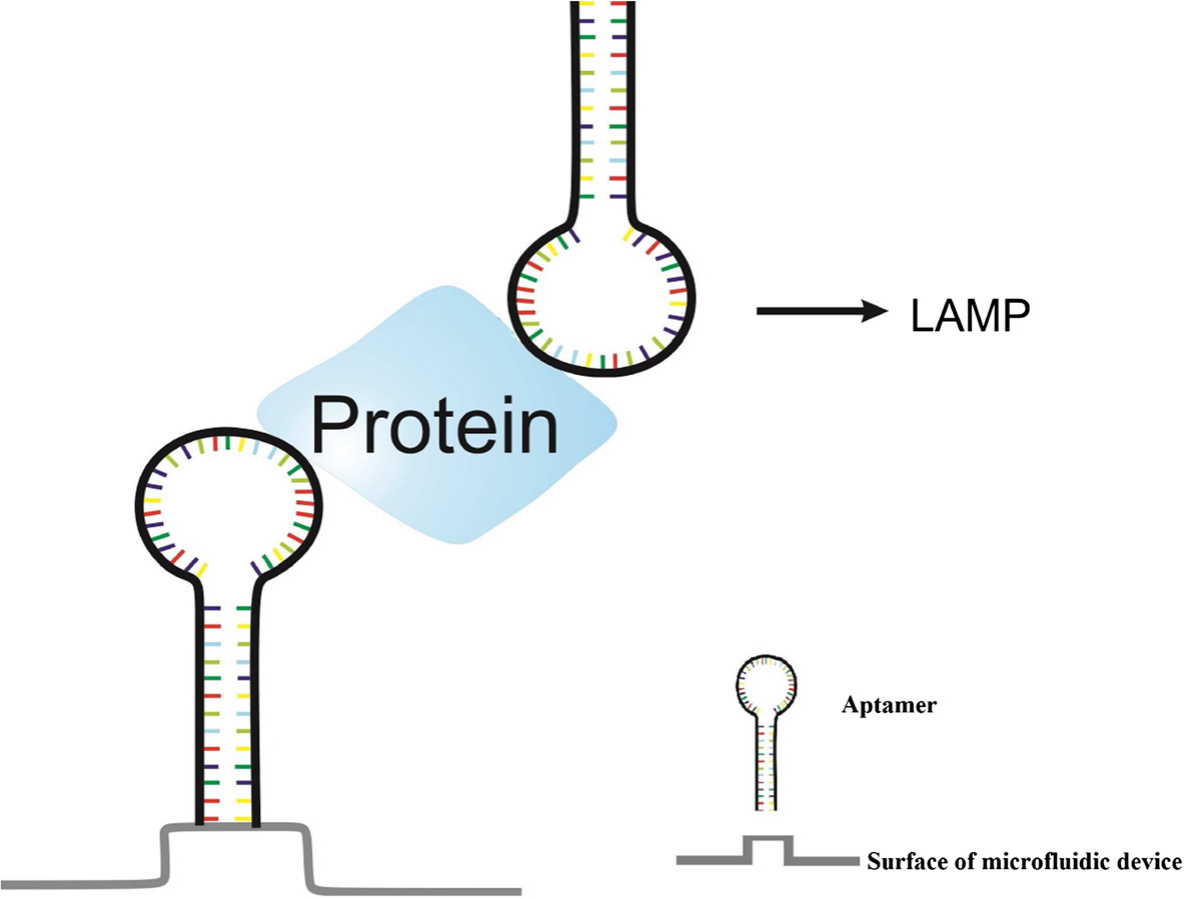
Integration of microfluidics with iLAMP (microfluidics-iLAMP platform).

Microfluidics, the handling of fluids in the micro/nanoscale, is an evolving field of analytical sciences, which allow precise control of fluid behavior under controlled conditions [57]. This precise control of fluids represents microfluidic-based devices as an advanced tool for analysis of biological samples. In fact, such devices have advantages that offer greater potential for the diagnostic tests to be practical for the clinical purposes. These advantages include the ability to handle small sample sizes with minimal reagents, the potential applications in field use or point-of-care (POC) usage, the possibility of integrating with biosensors or sample-pre-treatment modules, which increases the efficiency of the assays and reduces cross-contamination, decrease in the cost of test and short assay time [58,59]. Due to the advantages of microfluidic devices in the design of diagnostic methods, the integration of microfluidic-based devices with iLAMP increases its applicability in the clinical area. The scheme of microfluidic chip-based iLAMP platform is depicted in Figure 4.

#### Integration with aptamer (aptamer-iLAMP)

One of the challenges of current nucleic acid-based detection of proteins is the availability of antibody-signal DNA conjugates. In practice, the preparation of such conjugates is challenging, and sometimes the produced conjugates do not have the required specificity and produce background noise [20]. Due to the application of such conjugates in iLAMP, this problem also remains in iLAMP method. However, this problem can be solved by application of aptamers, the nucleic acids with the ability of specific recognition of their target molecules in the folded conformation instead of antibodies in iLAMP. Beside this advantage, aptamers have many benefits over antibodies. These advantages include low cost, ease of preparation, high stability, the possibility of reversible denaturing, the possibility of on-demand changes of the properties, the possibility of aptamer identification for toxins as well as molecules that do not elicit good immune responses, the minimum batch-to-batch difference in the performance, and the possibility of precise conjugation with various reporter molecules [60,61]. Aptamers also have high sensitivity and specificity toward their targets. They have the equilibrium dissociation constants (Kd) of the pico- to micromolar range and can discriminate subtle differences in the structure of their targets, even better than antibodies [62,63], while some disadvantages can limit the application of antibodies. Laborious production, limited shelf-life, restriction in the production of different types of antibodies due to the limitations in the growth of some hybridomas, batch-to-batch differences in the performance of the same antibody, and inability to modify the kinetic parameters of antibody-target interactions are the main drawbacks of the use of antibodies [63].

**Figure 5:**
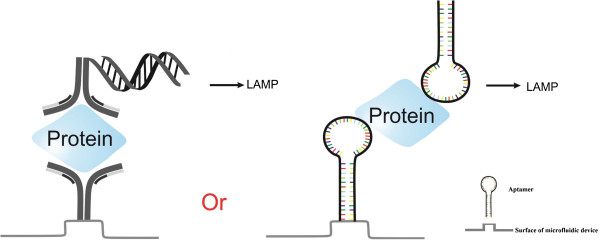
Application of aptamer instead of antibody in iLAMP (aptamer-iLAMP platform).

Based on the advantages of aptamers over antibodies, aptamer-iLAMP method can be considered as an improved configuration of iLAMP technique. In addition, the aptamers used can serve as both recognition and signal molecule for direct detection of the target without need for antibody-DNA conjugates (Figure 5).

### Possible limitations of iLAMP and their solutions

Like any new detection method, iLAMP may have some potential problems. One problem can be the challenging preparation of antibody-DNA conjugates. As discussed in the subsection ‘Integration with aptamer (aptamer-iLAMP)’ this problem can be solved by using aptamer molecules instead of antibody-DNA conjugates. In this case, aptamer can be used both for recognition and as a substrate of signal amplification (Figure 5).

The second problem may be related to the difficulty in the designing of LAMP primers. This problem can be alleviated by using a special software, called Primer Explorer (primerexplorer.jp/e/), which is designed specifically for LAMP primers.

Another problem may be related to the preparation of gold and silver nanoprobes. This step may add some complicacy in the procedure of protein detection with iLAMP-nanoprobe method. However, if the same DNA signal is used for different protein targets, the nanoprobes are the same for different proteins. This can lower the need for preparation of new nanoprobes for every protein target.

## Importance of the hypothesis

The proposed method can find various applications in the field of protein detection science. Due to ultra-high specificity and sensitivity of iLAMP, it can be used for detection of proteins with ultra-low concentrations (hardly detectable with common immunoassay methods), which is of high importance. These proteins include cancer biomarkers, viral proteins, toxins, hormones, allergens, pollutants, and small non-protein molecules (can be detected by aptamer-LAMP version) [20].

The proposed method can also be used for the detection of the surface antigens of different cells. In this case, particular antigens can be used to specifically detect the target cells for various purposes. Stem cells, rare circulating cells, such as circulating tumor [64] and fetal cells [65], and different subtypes of particular cells [66] can be easily detected using different configurations of iLAMP.

**Figure 6:**
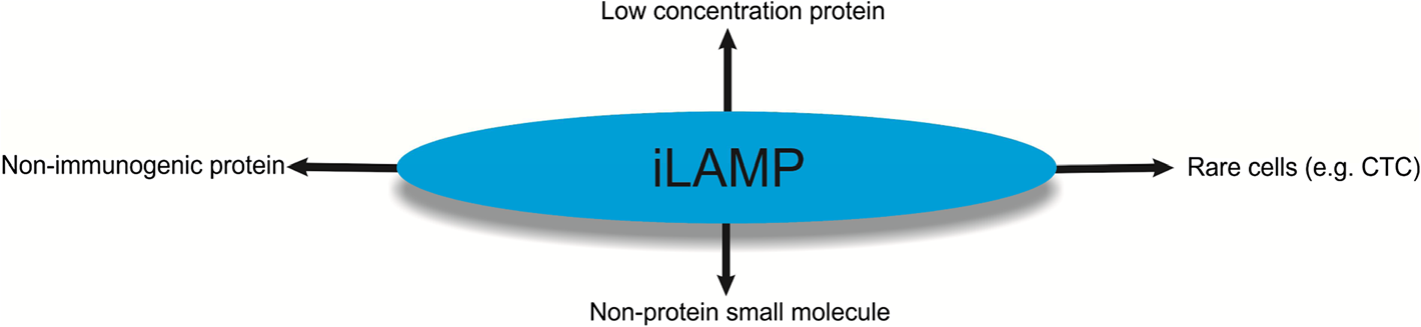
Possible applications of iLAMP technique.

The ultra-high sensitivity and specificity of iLAMP method allows one to identify many diseases as early as possible. This issue has a great importance in the case of lethal diseases like cancer due to the fact that early detection can increase the chance of successful treatments [67] (Figure 6).

## Summary and future perspectives

With the application of iLAMP method, many technical problems of current nucleic acid-based methods for protein detection can be avoided. This new method thus can find many potential applications in detecting low-concentration proteins that are vital for monitoring human diseases and pathological states in the human body. In conclusion, considering the rapidness, simplicity, and affordability with no need for expert personnel and specific instrument, iLAMP method can be an important alternative in point-of-care diagnostic technique, particularly in low-resource laboratories.

## Competing interests

The authors declare no competing interests.

## Authors' contributions

MPM designed the nanoprobes-related part of the study, did the literature review, and drafted the paper. MRY helped in the designing of the study and prepared the introduction section. AA designed the liposome-related part of the study and helped in drafting the paper. HD designed the aptamer-related part of the study and helped in drafting of the paper. KNK designed microfluidic-related part of the study. YH compared the literature review results. SWJ revised the paper and edited English writing of the paper. All authors read and approved the final manuscript.
